# The *yellow* gene regulates behavioural plasticity by repressing male courtship in *Bicyclus anynana* butterflies

**DOI:** 10.1098/rspb.2021.2665

**Published:** 2022-04-13

**Authors:** Heidi Connahs, Eunice Jingmei Tan, Yi Ting Ter, Emilie Dion, Yuji Matsuoka, Ashley Bear, Antónia Monteiro

**Affiliations:** ^1^ Department of Biological Sciences, National University of Singapore, 14 Science Drive 4, Singapore 117543; ^2^ Yale-NUS College, 16 College Avenue West, Singapore 138527; ^3^ Department of Ecology and Evolutionary Biology, Yale University, CT 06511, USA

**Keywords:** transcriptomics, 20-hydroxyecdysone, *yellow*, transgenic knock-out, courtship plasticity, butterfly

## Abstract

Seasonal plasticity in male courtship in *Bicyclus anynana* butterflies is due to variation in levels of the steroid hormone 20E (20-hydroxyecdysone) during pupation. Wet season (WS) males have high levels of 20E and become active courters. Dry season (DS) males have lower levels of 20E and reduced courtship rates. However, WS courtship rates can be achieved if DS male pupae are injected with 20E at 30% of pupation. Here, we investigated the genes involved in male courtship plasticity and examined whether 20E plays an organizational role in the pupal brain that later influences the sexual behaviour of adults. We show that DS pupal brains have a sevenfold upregulation of the *yellow* gene relative to the WS brains, and that knocking out *yellow* leads to increased male courtship. We find that injecting 20E into DS pupa reduced *yellow* expression although not significantly. Our results show that *yellow* is a repressor of the neural circuity for male courtship behaviour in *B. anynana*. 20E levels experienced during pupation could play an organizational role during pupal brain development by regulating *yellow* expression, however, other factors might also be involved. Our findings are in striking contrast to *Drosophila* where *yellow* is required for male courtship.

## 1. Introduction

Behavioural plasticity is essential for animals to adapt to environmental variation and it is often triggered by hormonal changes that organize or activate neural circuits in the brain [1–3]. Seasonal changes in temperature and photoperiod can serve as important cues that alter hormone levels and sexual behaviour in a wide range of vertebrate and invertebrate taxa [4]. Precisely how hormone signalling influences sexual behaviour and the identity of the downstream genes in most animals however, is not well known [5]. In vertebrates, hormones are considered to play both a brain organizational role during development, as well as a behavioural activational role in adults, compared to just an adult activational role in insects [6,7]. Whether hormones also display a similar latent role in insects remains unclear [7].

Most research on the genetics of courtship behaviour is based on studies in *Drosophila* [8,9]. Among the most well-studied genes are the transcription factors *fruitless* (*fru*) and *doublesex* (*dsx*), which undergo alternative splicing to produce male-specific proteins [10]. One of the genes proposed to be downstream of *fru*^M^ is *yellow,* a gene that is unique to insects and appears to have numerous biological functions, including melanin pigmentation, cuticle formation, butterfly scale colour and morphology and also male sexual behaviour [9,11–15]. Exactly how *yellow* expression influences male courtship behaviour in *Drosophila* has been an important topic of investigation that has yielded conflicting results. Early studies suggested that *yellow* male mutants were less successful during courtship due to neural circuitry regulation of courtship song [8,9,16]. More recent work demonstrated that *yellow* does not influence male courtship through any neural circuitry but rather by influencing the mechanical structure of the male sex combs [15]. These findings, therefore, contradict the long-held assumption that *yellow* is part of the neural circuitry governed by *fru*^M^ that regulates male courtship behaviour.

Sexual behaviour in insects has traditionally been viewed as a consequence of cell-autonomous processes taking place during brain development, and involving sex determination genes [6] such as *fruitless* and *doublesex* [17]. The role of insect hormones is typically described as playing an activational role, allowing rapid and reversible behavioural changes, such as activating neural circuits that regulate pheromone communication or sexual receptivity [18–21]. However, hormones have also been proposed to play an organizational role in insects, for instance in the regulation of behavioural polyphenisms in honeybees and locusts [7] and sexual maturity in *Drosophila* [22]. Yet, no evidence is available for the organizational role of steroid hormones in driving sexual behaviours in adult insects, similar to the latent role of steroid hormones in vertebrate sexual differentiation, where exposure to different hormone levels during ontogeny leads to discrete, fixed differences in neural development and sexual behaviours [7].

For insects living in seasonal environments, hormones could play an organizational role earlier in development to ensure that sexual behaviour is optimized for particular environmental conditions that will be prevalent upon adult emergence [23]. An example of a species where such a brain organizational role may be happening is the African seasonal polyphenic butterfly, *Bicyclus anynana*. This species shows an interesting sex-role reversal between seasonal forms that develop at different temperatures, and where temperature cues in the arrival of different seasons. In particular, wet season (WS) males, reared at high temperatures, play the active courting role, while dry season (DS) males, reared at low temperatures court less and become the choosy sex [24]. The adaptive reason driving courtship plasticity in males is associated with increased reproductive costs for DS males, which provide females with beneficial spermatophores [24]. Provision of this spermatophore ultimately shortens DS male lifespan, but lengthens DS female lifespan and helps them survive through the more stressful and resource-limited DS [23,24]. While the behavioural ecology of these butterflies may explain seasonal variation in male courtship rates, the neural re-wiring that switches the male behaviour is completely unknown.

In *B. anynana*, signalling of the hormone 20-hydroxyecdysone (20E) during early pupal development has been shown to regulate male courtship [25]. Throughout pupal development, there are significantly lower levels of 20E titres circulating in the haemolymph of DS than of WS males [25]. The reduced courtship of DS males, however, can be switched to the WS active courting form by rearing pupae at higher temperatures during the first 50% of pupal development [23] or, alternatively, by keeping the pupae at low temperatures but injecting them with 20E at 30% of pupal development [25]. These experiments suggest that this pupal stage is a critical period that determines male sexual behaviour and that 20E may play an organizing role in the developing male brain of *B. anynana*. However, we currently have no direct evidence that 20E acts specifically on the brain to alter expression of genes that regulate male courtship behaviour in adults. High levels of 20E could upregulate genes required for active courtship such as those described for *Drosophila* including *fruitless, doublesex* and *yellow* [9,15,17]. Alternatively, 20E signalling may not lead to appreciable differences in the neural circuitry of dry and WS male brains, but may instead influence other phenotypic traits that are also important in courtship behaviour such as pheromone production [26] or the UV brightness of eyespot centres [24].

We investigated whether genes are differentially expressed in DS and WS male brains at 30% of pupal development, and which genes, if any, are responding to 20E injections at that same time period. We conducted a transcriptome analysis on dissected brains of vehicle-injected DS and WS forms, and brains of DS forms injected with 20E, all at 2 h after the injection. Our transcriptome analysis revealed that the *yellow* gene is differentially expressed between seasonal forms. We then pursued additional qPCR experiments and functional studies by knocking out *yellow* using CRISPR-Cas9 and observing the courtship behaviour of mutant and wild-type males.

## 2. Material and methods

### (a) Transcriptome assembly and analyses

To mimic DS and WS conditions, caterpillars of *B. anynana* (established in New Haven, CT, from an original Malawi stock collected in 1988) were reared under WS and DS temperatures in climate-controlled rooms at 27°C and 17°C, respectively, at 80% humidity, and a 12 : 12 h light : dark photoperiod. Caterpillars were fed corn plants ad libitum until pupation. Pupae were staged, such that the per cent of pupal development was known for all individuals. At 30% of pupal development (day 2 in the WS butterflies and day 6 in the DS butterflies) DS pupae were injected with either 3 µl of 2000 pg µl^−1^ (6000 pg total) (10% 20E in EtOH + 90% saline) of 20E (Sigma-Aldrich) or with 3 μl of vehicle (10% EtOH and 90% saline) and WS pupae were injected with 3 μl of vehicle in the lateral posterior region of the fifth abdominal segment. The injections were done at 12.00 h and the brains were dissected 2 h later, at 14.00 h. We injected the pupae 2 h before brain collection because previous studies have demonstrated that genes, which respond early to 20E signalling, are expressed about 2 h after exposure to 20E [27,28].

Each sample consisted of three biological replicates of WS pupal brains following treatment with vehicle only (WSV); four biological replicates of DS pupal brains treated with vehicle only (DSV); and five biological replicates of DS pupal brains treated with 20E (DS20E). Each biological replicate contained the brains of five male pupae. We assembled the transcriptome using 12 RNA-Seq libraries. Details of the sample preparation and transcriptome assembly can be found in the electronic supplementary material, Methods section.

The differentially expressed genes (DEGs) were annotated with Blast2GO v. 5.2.5. We used the public NCBI Blast service (QBlast) to blast our sequences against the non-redundant protein database using the blastx-fast program. Matched transcripts were filtered using a cut-off *E*-value of 1 × 10^−3^; otherwise the default settings for Blast2GO were used at each step. To annotate the remaining transcriptome, we performed a local blastx of the assembled contigs against the *B. anynana* v. 1.2 draft genome [29].

### (b) qPCR sample collection and experiments

Sample collection was similar to that described above for the RNA-Seq experiment (see electronic supplementary material, Methods section and table S1). At 30% development, we injected pupae with 20E or vehicle solutions using the same protocol as described above. Pupae were kept in their respective development temperature and brains were dissected 2 h, 4 h and 24 h after injections in ice cold 1× PBS, placed immediately into RNALater (Qiagen, GmbH, Hilden, Germany) and stored at −20°C until RNA extraction. We used five biological replicates per treatment, each made of five pooled brains (for the 2 h dissections) or two pooled brains (for the 4- and 24-hour dissections).

### (c) Behavioural assays

We conducted behavioural assays using a Yellow mutant line to investigate whether loss of *yellow* function affects butterfly courtship behaviour. To establish the Yellow mutant line, we inserted an attP sequence into exon 4 of the *yellow* gene to disrupt its overall sequence (electronic supplementary material, figure S1). We used a knock-in method through homology-directed repair (HDR) using a single-stranded DNA (ssDNA) as a template. The ssDNA construct was made following methods described in [30]. Further details are in the electronic supplementary material, Methods section.

Behavioural assays were conducted in cylindrical hanging cages (30 × 40 cm) under one full-spectrum light tube (Plantmax) and one UV light bulb (Arcadia Marine Blue), at 23°C, from 17.00 to 18.00. This specific time of observation was chosen because *B. anynana* exhibits crepuscular courtship [25]. Visual barriers were placed between cages to prevent mate-copying [31]. Within each sex, butterflies used for each assay were of the same age. All butterflies used in the assays ranged from 4 to 8 days old. Two experiments were performed, one with live females and the other with decapitated live females. The treatments were (i) two Wt males × two Wt females and (ii) two Yellow mutant males × two Wt females (electronic supplementary material, figure S2). One of the two males/females in an assay was dotted with a black marker at both of its ventral hindwings to allow for sex-specific scoring of behaviour. The multiple elements of courtship, as documented in Nieberding *et al*. [32], were scored in the assays: (1) localization (flying to other butterfly), (2) rapid flickering of wings, (3) thrusting (touching female's wings with head) and (4) attempting (curling of the abdomen). Orientation (orienting body to female's posterior) was not recorded since it was difficult to score or interpret their intent (courtship or coincidence) with that behaviour. Latency to mate (time taken from the start of assay to the first mating) and mating duration were recorded as well. Behavioural assays lasted 1 h, and quantification of an individual male's behaviour stopped once it mated but the other male's behaviour was still quantified until its own mating, or 1 h, had lapsed).

For decapitation assays, only females were decapitated to characterize male sexual behaviour in the absence of female response [33]. Decapitated females were first anaesthetized in a −20°C freezer for 20 min and their heads were removed. Females were pinned through their thorax into opposite sides of the cage as illustrated in electronic supplementary material, figure S2c,d. The same behavioural scoring as described above was used for the decapitation assays as well.

### (d) Statistical analysis

The data were evaluated for equality of variances and normality using the Levene's test and Shapiro–Wilk test, respectively. Total duration and frequency of courtship were calculated by adding up the duration/ frequency of all the courtship elements displayed (localizing + flicker + thrust + attempt) during the observation period. A generalized linear model (GLM) with a Tweedie distribution (gamma family; tweedie package [34]) was done to test the impact of the treatment type (Yellow/Wt) and season (WS/DS) on the total duration of courtship. A Tweedie distribution (gamma) was used due to the high number of zeroes and the skewness of data. The impact of the treatment type (Yellow/Wt) and season (WS/DS) on the frequency of each courtship element was compared using a zero-inflated Poisson model with a negative binomial distribution to account for overdispersion (pscl package) [35]. Both mating latency and mating duration of the mated pairs were compared using independent *t*-tests. χ^2^-tests were carried out to identify any associations between treatment type and mating success. Statistical tests and figures were done with IBM SPSS Statistics 25 and R-v. 4.0.2 [36]. The spectral data of eyespots were visualized using the pavo package (electronic supplementary material, figure S3 [37]). Spectral analysis was done through calculating area under curve (AUC) for each eyespot replicate and the AUC analysed using an ANOVA with *post hoc* Tukey test in R.

## 3. Results

**Figure 1.**
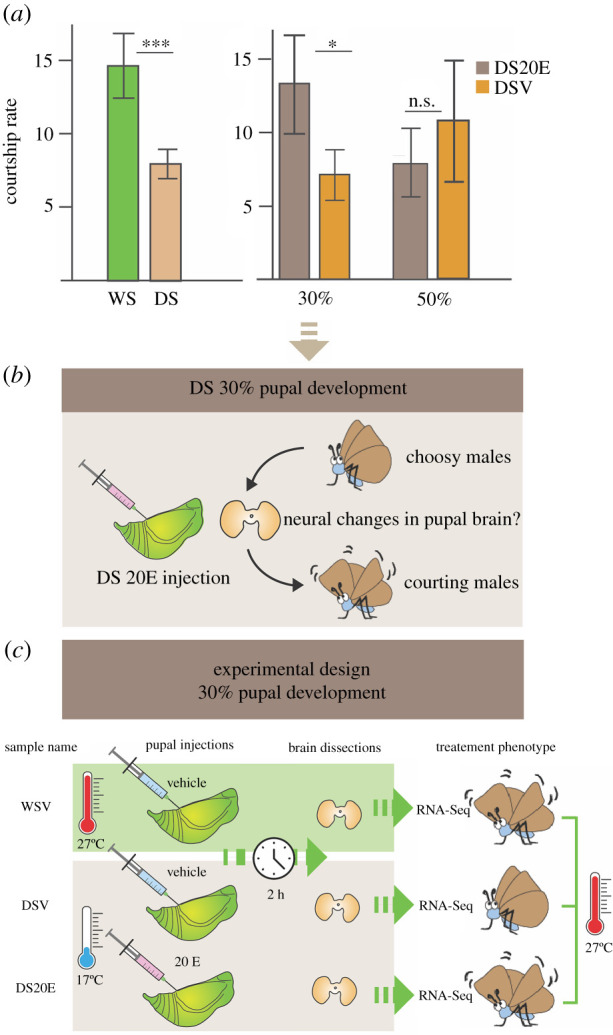
(*a*) Differences in male courtship rates between wet season (WS) and dry season (DS), DS injected with 20E (DS20E) versus DS injected with vehicle (DSV). DS20E shows similar courtship rates to WS but only when injected at 30% of pupation (adapted from [25]). (*b*) Schematic of our hypothesis that 30% of pupation represents a critical window in which levels of 20E can determine male courtship behaviour by causing organizational effects on neural circuitry in the developing pupal brain. (*c*) Overview of the experimental set-up for the pupal injections, brain dissections and RNA-seq for the three different treatment groups. (Online version in colour.)

The critical window for determining male courtship behaviour occurs at 30% of pupation, as both temperature shift experiments or injections of 20E into DS pupa at this specific stage, rescues WS courtship rates [25]. Therefore, we reasoned that temperature may be affecting 20E levels, and 20E may be affecting gene expression in the pupal brain causing a switch in adult behaviour from a choosy male to an active courter. To test this hypothesis, we compared the transcriptome of DS male brains injected with 20E at 30% pupal development with DS and WS male brains injected with a vehicle solution at the same developmental stage (DSV and WSV, respectively) to identify genes that may be regulating courtship behaviour (figure 1).

We identified 399 DEGs between DSV and WSV pupal brains, 302 were upregulated and 97 were downregulated in DSV. Comparing DS20E pupal brains to WSV, we identified 399 DEGs, 291 were upregulated and 108 were downregulated in DS20E. Comparing DS20E pupal brains to DSV we identified 151 DEGs, 79 were upregulated and 72 were downregulated in DS20E. Overall, the smaller number of DEGs observed between DSV and DS20E (151) compared to DSV and WSV (399) suggest that the DS20E brain transcriptome profile was more similar to DSV than WSV at 2 h post-injection (electronic supplementary material, figure S4). Full summary statistics are provided in electronic supplementary material, table S2 and figures S4–S5. The list of DEGs can be found in electronic supplementary material, tables S3–S4.

### (a) The *yellow* gene which drives male courtship in *Drosophila* was significantly upregulated in DSV and DS20E pupal brains

**Figure 2.**
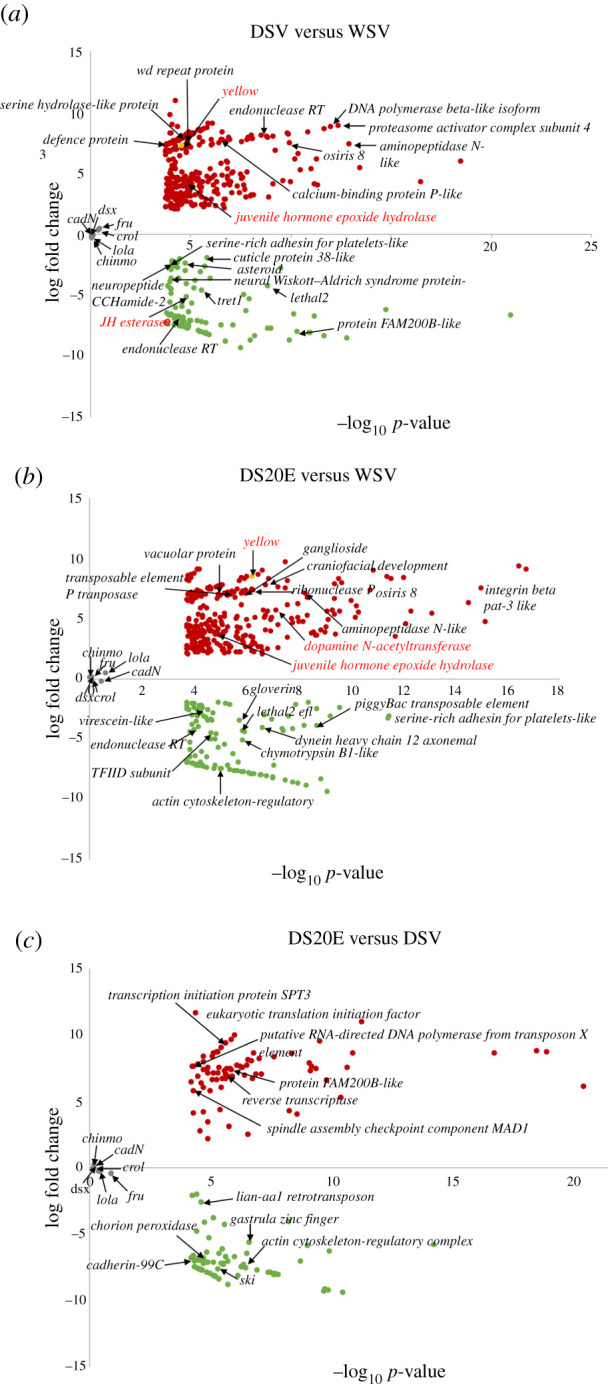
Volcano plots summarizing the log-fold change values and *p*-values of the differentially expressed genes (DEGs). Yellow gene was upregulated sevenfold in DSV and eightfold in DS20E. Annotations are shown for the top 10 DEGs which returned hits using the software Blast2Go (blastx to NCBI). Dopamine *N*-acetyltransferase (AANAT) and Juvenile epoxide hydrolase are also shown although they were not in the top 10. Upregulated genes are shown in red (positive values) and downregulated genes are shown in green (negative values). (*a*) Genes up- and downregulated in brains from the dry season vehicle treatment compared to the wet season vehicle treatment (DSV versus WSV). (*b*) Genes up- and downregulated in brains from the DS20E treatment (dry season pupa injected with 20E) compared to the WSV treatment (DS20E versus WSV). (*c*) Genes up- and downregulated in brains from the DS20E treatment compared to DSV (DS20E versus DSV). Genes involved in male courtship behaviour in *Drosophila* that were not differentially expressed in any comparison (grey). See electronic supplementary material for details of log fold changes. (Online version in colour.)

The melanin pathway gene *yellow* was significantly upregulated in both DSV (sevenfold increase) and DS20E (eightfold increase) when compared to WSV. Other genes of interest pertaining to courtship behaviour included genes involved in the Juvenile hormone signalling pathway, such as *Juvenile hormone esterase* (JHE), which was downregulated in DSV compared to WSV, and *Juvenile hormone epoxide hydrolase-like* (JHEH, which hydrolyses JH) which was upregulated in DSV and DS20E when compared to WSV. We also identified a gene involved in dopamine metabolism, *dopamine N-acetyltransferase* (AANAT1/DAT1) which was upregulated in DS20E (but not in DSV) when compared to WSV. Genes involved in neural development included *neuropeptide CCHamide2, Neural Wiskott–Aldrich syndrome protein* and *lethal 2 essential for life l(2)efl* (electronic supplementary material, table S4). These genes were downregulated in DSV compared to WSV with *l(2)efl* also downregulated in DS20E compared to WSV. Genes known to be important in male courtship behaviour such as *fruitless* and *doublesex* [10,38] were not differentially expressed at 30% of pupal brain development (figure 2).

### (b) 20E downregulates the expression of *yellow* 4 h after injections

*Yellow* is an interesting candidate to explore further due to its importance for male courtship in *Drosophila* [9,15]. As the transcriptome showed that it was significantly upregulated in both DS treatments compared to WS, we considered whether the 2-h time point for brain dissections was too early to detect any effects of 20E on *yellow* expression. We hypothesized that injection of 20E into DS male pupae would reduce *yellow* expression down to WS levels at a later time point post-injection. Similar to the transcriptome experiment, we injected 20E in DS male pupae at 30% development [25] and a vehicle solution in both DS and WS pupae at the same stage, and used qPCR to measure expression of *yellow* in dissected pupal brains at 2, 4 and 24 h post injection.

**Figure 3.**
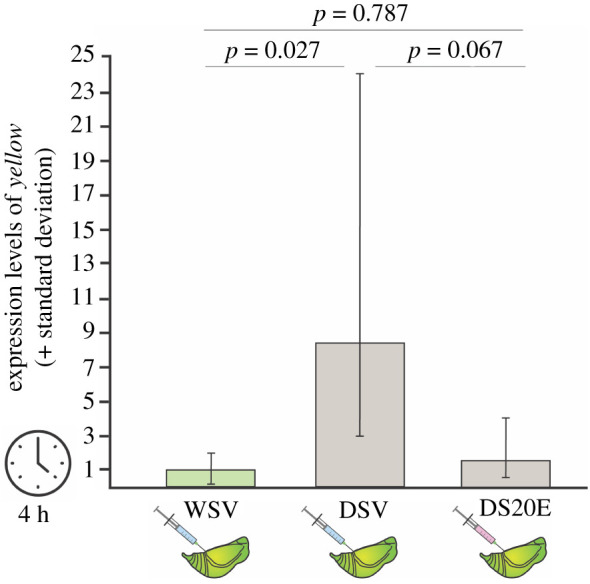
Four hours after injections, *yellow* is downregulated in brains of DS pupae injected with 20E compared to pupae injected with vehicle. Bars show fold change expression relative to WS pupae injected with vehicle solution. Indicated *p*-values are the Turkey-adjusted *p*-values from the *post hoc* analysis. (Online version in colour.)

Two hours post-injection, *yellow* expression was 2.5 times higher in pupal brains of both DSV and DS20E compared to the expression in WSV pupal brains, (mirroring our RNA-seq results), although the expression levels were not significantly different (electronic supplementary material, figure S6, ANOVA: *F* = 0.50, *p* = 0.62). At 4 h post-injection, expression of *yellow* increased significantly by eightfold in DSV compared to WSV pupal brains (figure 3, ANOVA: *F* = 5.43, *p* = 0.023; *post hoc* analysis WSV-DSV: adj. *p* = 0.027), while the level of *yellow* expression in DS20E remained low, similar to those of WSV brains (ANOVA *post hoc* analysis WSV-DS20E: adj. *p* = 0.79). At 24 h post-injection, expression levels of *yellow* in the pupal brains were 2.8 (DSV) and 3.7 (DS20E) times higher than in WSV (electronic supplementary material, figure S6). Relative levels of *yellow* expression were significantly higher in DS20E than in WSV pupal brains (ANOVA: *F* = 4.12, *p* = 0.046; *post hoc* analysis WSV versus DS20E: adj. *p* = 0.046). These results demonstrate that a single injection of 20E into DS pupae at 30% of development was sufficient to decrease *yellow* expression levels in DS20E to WSV levels at 4 h post-injection. This single injection did not impact *yellow* levels at the earlier 2 h time period, nor kept *yellow* levels low at 24 h post injection, suggesting that a short interval of time around 30% pupal development encompasses a hormone-sensitive window in which *yellow* is downregulated by 20E to WS levels.

### (c) Yellow males courted more frequently and for a longer duration than Wt males

**Figure 4.**
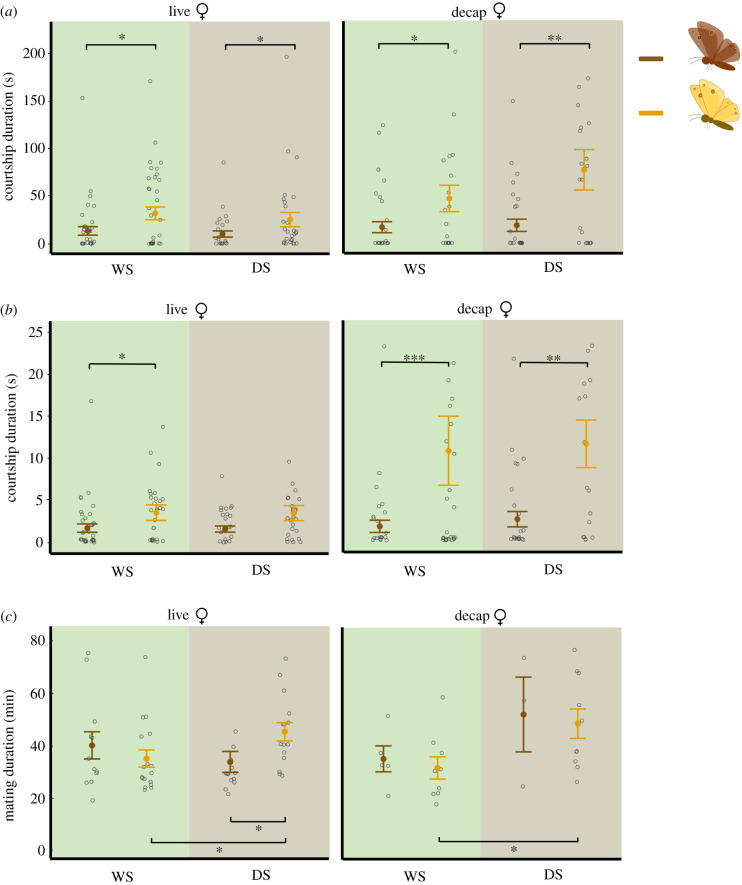
Courtship behaviour of Wt and Yellow males, for wet (WS) and dry (DS) seasonal forms, in both live and decapitation assays. Yellow males courted at a higher duration and frequency than Wt males, for both WS and DS forms. DS Yellow males remained in copula for a longer period of time than WS Yellow males. (*a*) Courtship duration, (*b*) courtship frequency and (*c*) mating duration were quantified. Mating duration was quantified among mated males only. Vertical bars represent mean + SEM. Open circles are data points. Asterisks indicate significant differences: *p ≤ 0.05, **p ≤ 0.01, ***p ≤ 0.001. Outliers are removed from the figures (Q1 – 3*1QR or Q3 + 3* IQR). n(WS-Live-Wt) and n(WS-Live-Yellow) = 38, n(WS-Decap-Wt) and n(WS-Decap-Yellow) = 34, n(DS-Live-Wt), n(DS-Live-Yellow), n(DS-Decap-Wt) and n(DS-Decap-Yellow) = 30. (Online version in colour.)

Based on the transcriptome and qPCR results we hypothesized that *yellow* is a repressor of male courtship as DS males exhibit lower courtship than WS males and have significantly higher expression of *yellow* during pupal brain development. Using a Yellow mutant line (hereafter Yellow males), we compared the duration and frequency of the Yellow males and the wild-type (Wt) male courtship sequence, including copulation, in both seasonal forms. These behavioural assays were initially performed with live females. Yellow males courted for a longer duration (WS: *t* = 2.181, *p* = 0.0323; DS: *t* = 2.083, *p* = 0.0416; figure 4*a*) than Wt males regardless of seasonality. Yellow males also courted more frequently (WS: *z* = 2.465, *p* = 0.0137; figure 4*b*) than Wt males, but this was only observed in the WS form. In addition, Yellow males remained in copulation longer with live females (*t* = 2.174, *p* = 0.039; figure 4*c*) than Wt males, but this was only observed in the DS form.

We found that both Yellow and Wt males had similar ultraviolet (UV) reflectivity in their dorsal eyespot centres—a known sexual ornament (electronic supplementary material, figure S3) [39,40]. Thus, females would not be choosing males based on their dorsal UV reflective eyespot centres. Yellow males displayed an overall lighter pigmentation compared to Wt males, which might influence female's receptivity to mating with a novel phenotype. Hence, we repeated these male courtship observations using freshly decapitated females. Decapitation prevents important visual cues detected by a female from impacting a male's behaviour, such as more intense courtship provoked by a female's increased rejection behaviour [33]. Yellow males still courted for a longer duration (WS: *t* = 2.269, *p* = 0.0266; DS: *t* = 3.21, *p* = 0.00217; figure 4*a*) and more frequently (WS: *z* = 3.423, *p* = 0.00062; DS: *z* = 3.2, *p* < 0.00137; figure 4*b*) than Wt males regardless of seasonality. This result indicates that Yellow alters male courtship behaviour independently of the female's behaviour toward those males.

### (d) Yellow males copulated longer in their DS form than WS form

To test whether differences in *yellow* expression levels were sufficient to explain courtship differences between the seasonal forms, we compared the duration and frequency of courtship between DS and WS Yellow males. If Yellow, alone, was responsible for courtship differences between the forms, then DS and WS Yellow males should display similar levels of courtship. There were no differences in the duration or frequency of courtship between DS and WS Yellow males (figures 4*a* and *b*). However, there was a significant difference in mating duration between the Yellow seasonal forms. With live females, DS Yellow males copulated longer (*t* = −2.119, *p* = 0.0425; figure 4*c*) than WS Yellow males. Similar behaviour was observed in males courting decapitated females, with DS Yellow males copulating longer (*t* = −2.34, *p* = 0.0318; figure 4*c*) than their WS counterparts.

## 4. Discussion

In insects, hormones are typically assumed to regulate sexual behaviour by activating existing neural circuits that control processes such as sexual maturation, memory formation and pheromone communication [19,41–43]. It remains unclear, however, whether insect hormones also exhibit a latency effect whereby exposure earlier in development can organize neural circuits that affect sexual behaviour in adults. The identity of these hormone-sensitive genes involved in neural circuits regulating male courtship also remains unknown. Although, we did not find conclusive evidence that 20E plays an organizational role, we show that *yellow* is involved in regulating seasonal courtship plasticity by repressing male courtship behaviour.

### (a) Yellow functions as a repressor of male courtship in *B. anynana*

We show, using RNA-seq, that *yellow* is significantly upregulated by sevenfold in the pupal brains of DS male butterflies which court less than WS males. This increase in *yellow* expression could be in response to seasonal fluctuations in 20E, as injection of this hormone into DS males at 30% of pupal development rescues WS courtship levels [25]. Our qPCR results for 4 h post-injection of 20E into DS pupa, showed that while levels of *yellow* expression declined to similar levels observed in WS males, the difference was not significant from DS levels. High levels of *yellow* in DS males suggested that *yellow* was a repressor of courtship. This was confirmed by knocking out *yellow* in *B. anynana* and observing Yellow males exhibiting increased courtship frequency and duration compared to Wt males of both seasonal forms. Given that Yellow WS males displayed more active courtship than Wt WS males, this suggests that low levels of *yellow* expression are still required in Wt WS males to reduce courtship and optimize energy expenditure, as increased wing fluttering observed in Yellow males did not translate to increased mating success (electronic supplementary material, figure S7).

Our findings are in striking contrast to those observed in *Drosophila* where *yellow* is required for normal male courtship behaviour and male mating success. However, in *B. anynana* males, *yellow* functions as a repressor of male courtship and does not disrupt mating success. Early studies in *Drosophila* suggested that *yellow* mutant males were less active during courtship and that mutations in *yellow* disrupted wing extension during the courtship ritual, preventing males from performing a courtship song which is required for male mating success [9,10]. However, recent work by Massey *et al*. argued that a lack of melanization in the sex combs of *yellow* mutants, rather than any impairment in neural circuitry affecting courtship song, was the trait that prevented males from successfully grasping females [16]. All research to date on *yellow* mutants in *Drosophila,* however, clearly demonstrate that *yellow* is absolutely required for successful male courtship and mating success.

In *B. anynana*, it is possible that changes in *yellow* expression in tissues other than the brain might have influenced courtship behaviour of Yellow males. As butterflies do not have sex combs we can rule out this possibility. In the Yellow males, the white centre of the eyespot, which is crucial for courtship behaviour shows no difference in brightness between Yellow and Wt males (electronic supplementary material, figure S3) [39,40]. Thus, we conclude that our findings point toward *yellow* influencing neural circuits regulating behaviour.

In our experimental set-up, using two males and two females, we found no difference in courtship rates between DS and WS wild-type butterflies. This result conflicts with previous work showing that WS males court more than DS males in larger social groups [24,25]. This difference may be explained by the reduction of sexual competitors in our assay, previously shown to increase the likelihood of courting and copulating by DS males in this species [44].

### (b) Yellow may influence courtship behaviour in *B. anynana* via the dopaminergic signalling pathway

Our findings raise questions about how *yellow*, a gene involved in melanization, might regulate courtship behaviour. Melanin synthesis enzymes are expressed in the *Drosophila* brain and may be involved in the production of neuromelanin in dopaminergic neurons [45]. Yellow is thought to function as a dopachrome conversion enzyme (DCE) in the melanin pathway converting l-Dopa to Dopa-melanin [11,12]. l-Dopa is also used as a substrate for dopamine which is involved in both cuticle pigmentation and neurotransmission [46]. Dopaminergic signalling has been shown to regulate mating drive and persistence and duration of mating in male *Drosophila* [46,47]. Thus, variation in *yellow* expression could alter the availability of l-Dopa for dopamine synthesis, with higher expression of *yellow* in DS brains leading to reduced l-Dopa. Alternatively, Yellow may physically bind to dopamine, as demonstrated in a study of salivary proteins in sandflies [48]. Thus, increased expression of *yellow* in DS brains could lead to reduced dopamine availability, which may inhibit courtship behaviour. In electronic supplementary material, figure S8, we suggest a possible mechanism of Yellow involvement in the pathway converting tyrosine to l-Dopa in dopaminergic neurons.

Currently, we have no direct evidence that dopamine levels differ between DS and WS *Bicyclus* brains. However, a few genes from our transcriptome analyses may provide some indirect evidence. We found that dopamine *N*-acetyltransferase, AANAT/DAT1 was upregulated in pupal brains of DS20E (but not in DSV) as compared to WSV brains. This may indicate a transient response to the 20E injection. The function of AANAT is to metabolize and inactivate secreted dopamine in the synapse shortly after release [45,49]. In young female *Drosophila virilis*, higher titres of 20E lead to an increase in dopamine, although this appears to be associated with reduced activity of AANAT [50]. However, in retinal cells of fish, AANAT activity is positively correlated with dopamine levels [51]. An increase in dopamine induced by 20E would provide a mechanistic explanation as to why DS male pupa injected with 20E display active WS courtship behaviour.

We also observed changes in Juvenile hormone (JH) signalling, which is known to interact with dopamine to affect sexual maturity and courtship behaviour in *Drosophila,* likely through changes in neural development [22,52]. In DS pupal brains, JHE was downregulated and Juvenile hormone epoxide hydrolase (JHEH), which degrades JH [53], was upregulated. These findings could indicate that dopamine levels are low in DS male pupal brains as dopamine increases JH titres in young female *D. virilis* by inhibiting its degradation [54]. JH is also associated with increased dopamine levels in male honeybees [55]. Although we also see an upregulation of JHEH but no longer a downregulation of JHE in DS20E, this may reflect a response to changing levels of dopamine. Interactions between 20E, JH, dopamine and AANAT in *Drosophila* represent a complex pathway as depicted in [50,56] thus, we must interpret our findings with caution. However, given that this is an important pathway for regulating courtship behaviour in *Drosophila*, differential expression of these genes in our transcriptome analyses suggest their possible involvement in regulating courtship behaviour in *B. anynana*. Surprisingly, genes important for male courtship in *Drosophila* (*fruitless, dsx crol, lola, cadN* and *chinmo*) [38], all showed very low levels of expression and were not differentially expressed in *B. anynana* male brains (figure 2).

## 5. Conclusion

Here, we provide the first evidence for a gene regulating butterfly courtship behaviour. In *B. anynana*, *yellow* functions as a repressor of male courtship thus higher expression of this gene in DS pupal brains may explain why these butterflies court less than WS males. It remains unclear, however, why injection of 20E into DS pupae at 30% development converts their behaviour to the WS form. Further work is required to confirm whether 20E plays a role in organizing neural circuits (potentially involving *yellow*) during critical windows of brain development. Our results also suggest possible interactions of 20E on JH and dopamine signalling, a circuit well described in *Drosophila*. Future studies examining dopamine levels between the seasonal forms and the individual role of 20E and JH on dopaminergic signalling would help clarify mechanistically why the *yellow* gene functions as a repressor of male courtship in these butterflies. For animals living in seasonal environments, selection may favour adaptations that use external cues to optimize behaviour, such as employing environmentally induced hormones, even in insects.

## Data Availability

Raw reads of the RNA-seq libraries were uploaded to the SRA database with the SRA accession no. PRJNA544388. This Transcriptome Shotgun Assembly project has been deposited at DDBJ/EMBL/GenBank under the accession no. GHRJ00000000. The version described in this paper is the first version, GHRJ01000000. The annotated transcriptome is available from the Dryad Digital Repository: https://doi.org/10.5061/dryad.9cnp5hqg4 [57]. The data are provided in electronic supplementary material [58].
